# Antisense Oligonucleotide-Mediated Exon-skipping Therapies: Precision Medicine Spreading from Duchenne Muscular Dystrophy

**DOI:** 10.31662/jmaj.2021-0019

**Published:** 2021-07-09

**Authors:** Masafumi Matsuo

**Affiliations:** 1 KNC Department of Nucleic Acid Drug Discovery, Department of Physical Rehabilitation and Research Center for Locomotion Biology, Kobe Gakuin University, Kobe, Japan

**Keywords:** exon skipping, antisense oligonucleotide, reading frame, treatment, Duchenne muscular dystrophy

## Abstract

In 1995, we were the first to propose antisense oligonucleotide (ASO)-mediated exon-skipping therapy for the treatment of Duchenne muscular dystrophy (DMD), a noncurable, progressive muscle-wasting disease. DMD is caused by deletion mutations in one or more exons of the *DMD* gene that shift the translational reading frame and create a premature stop codon, thus prohibiting dystrophin production. The therapy aims to correct out-of-frame mRNAs to produce in-frame transcripts by removing an exon during splicing, with the resumption of dystrophin production. As this treatment is recognized as the most promising, many extensive studies have been performed to develop ASOs that induce the skipping of *DMD* exons. In 2016, an ASO designed to skip exon 51 was first approved by the Food and Drug Administration, which accelerated studies on the use of ASOs to treat other monogenic diseases. The ease of mRNA editing by ASO-mediated exon skipping has resulted in the further application of exon-skipping therapy to nonmonogenic diseases, such as diabetes mellites. Recently, this precision medicine strategy was drastically transformed for the emergent treatment of only one patient with one ASO, which represents a future aspect of ASO-mediated exon-skipping therapy for extremely rare diseases. Herein, the invention of ASO-mediated exon-skipping therapy for DMD and the current applications of ASO-mediated exon-skipping therapies are reviewed, and future perspectives on this therapeutic strategy are discussed. This overview will encourage studies on ASO-mediated exon-skipping therapy and will especially contribute to the development of treatments for noncurable diseases.

## Introduction

In 1995, we first proposed antisense oligonucleotide (ASO)-mediated exon-skipping therapy for Duchenne muscular dystrophy (DMD) ^(1), (2)^, and this method has since developed into a genuine strategy for DMD treatment. As exon-skipping therapy allows the editing of mRNA, it has been applied to treat both monogenic and nonmonogenic diseases. Remarkably, this precision medicine strategy was revolutionized for the treatment of a single patient, thus representing a future aspect of ASO-mediated exon-skipping therapy. In this article, ASO-mediated exon-skipping therapy is reviewed, beginning from its invention for DMD treatment to its wider uses in the treatments of nonmonogenic diseases, and future perspectives for ASO-mediated exon-skipping therapies are discussed.

## 1. Exon Skipping

Exon skipping involves the removal of certain exons from mRNAs during splicing, during which introns are removed from pre-mRNA to produce mature mRNA. Although splicing is strictly regulated to produce mRNAs with no errors, alternative splicing occurs in more than 90% of human genes to produce a variety of mRNAs and diverse protein products ^(3)^. Notably, exon skipping is the most common pattern of alternative splicing. In cancer cells, for example, 90,616 exon-skipping events are reported to occur in 14,272 genes ^(4)^.

On the other hand, exon skipping is induced by gene mutations and causes genetic diseases. Exons are defined by splicing consensus sequences located at the junction of exons and introns in pre-mRNA, and GT and AG dinucleotides are strictly conserved at the splice donor and acceptor sites, respectively. Furthermore, this definition is supported by the splicing cis elements located in exon or intron sequences, and most mutations that induce exon skipping are located at splicing consensus sequences. In addition, mutations splicing regulatory cis elements, such as exonic splicing enhancers/silencers and intronic splicing enhancers/silencers, cause exon skipping, and exon-skipping mutations are common in genetic diseases ^(5)^.

## 2. Duchenne Muscular Dystrophy

DMD (OMIM 310200), a fatal X chromosome-linked recessive disorder, is the most common inherited muscle disease and affects one in 5,000 to 6,000 live-born male children. DMD is characterized by infantile-onset progressive muscle wasting, and patients usually succumb to the disease during their 20s. Although DMD has remained uncurable since its initial appearance in 1868, the life expectancy of patients with DMD has been extended slowly by general medical treatments and physical therapy ^(6), (7)^, and establishing a fundamental treatment protocol has long been the goal of DMD studies.

In 1987, the *DMD* gene, responsible for DMD, was cloned ^(8)^ and shown to comprise 79 exons spanning 2.4 Mb on the X chromosome and to yield a 14-kb cDNA encoding dystrophin; dystrophin deficiency leads to DMD. Exon deletions, which are the most common deletion type (>50%), cause both DMD and Becker muscular dystrophy (BMD), a milder progressive muscle-wasting disease. The differences in the two types of diseases are attributed to a translational reading frame rule ^(9)^; mutations shifting the translational reading frame of the mRNA usually result in the more severe DMD phenotype because of the creation of premature stop codons that totally prohibit dystrophin protein expression, whereas mutations that maintain the original mRNA reading frame cause the milder BMD phenotype because a mutated but still functional dystrophin protein can be expressed from the mRNA.

## 3. Exon-skipping Therapy for DMD

### 3-1. Exon skipping in dystrophin Kobe

A case of a Japanese boy with DMD contributed greatly to the development of ASO-mediated exon-skipping therapy ^(10)^. For this patient, a small product of exon 19 of the *DMD* gene was produced by polymerase chain reaction (PCR), and the sequencing of the product revealed a 52-bp deletion within the 88-bp exon 19, thus shortening the exon to 36 bp ^(11)^. This out-of-frame genomic deletion in DMD is 6 bp upstream of the 3′ end of exon 19 and does not disrupt the splicing consensus sequences, consistent with the reading frame rule ^(9)^. This mutation caused the smallest deletion known at the time and was termed dystrophin Kobe ^(11)^.

Analysis of the patient’s *DMD* cDNA revealed a complete absence of the exon 19 sequence, indicating the skipping of exon 19, although the consensus sequences at the splicing acceptor and donor sites were intact. Remarkably, the cause of exon skipping was attributed to the deletion of an exonic splicing enhancer encoded by the deleted sequence ^(1)^. This was the first demonstration of an exonic splicing enhancer being critical for the recognition of *DMD* gene exons. Furthermore, ASOs directed against this splicing enhancer sequence were shown to inhibit the splicing of exon 19 ^(1)^.

### 3-2. Proposal of novel exon-skipping therapy

Based on the above findings, we proposed a novel precision medicine strategy for the use of ASO-mediated exon-skipping therapy for DMD. The induction of exon skipping allows one exon to be omitted from the mutated *DMD* mRNA, leading to the conversion of an out-of-frame mRNA to an in-frame mRNA, thereby restoring dystrophin production ^(1), (2)^. Subsequently, we showed that an ASO against exon 19 could induce the skipping of exon 19 of the endogenous transcript ^(12)^.

Furthermore, we obtained convincing evidence for the efficacy of exon-skipping therapy on a Japanese patient with BMD exhibiting a single-nucleotide mutation in exon 27 (c.3631G > T) that led to the substitution of glutamate for a stop codon (E1211X) ^(13)^. The single-nucleotide mutation induced the skipping of exon 27, thereby producing an in-frame semifunctional *DMD* mRNA sequence and leading to the production of a truncated dystrophin protein, with a mild disease phenotype. These results clearly indicate the potential effect of exon skipping as a treatment for DMD.

### 3-3. The first study on exon-skipping therapy

Theoretically, ASOs capable of inducing exon 19 skipping should produce in-frame mRNAs in patients with DMD carrying an exon 20 deletion. This was verified by the transfection of an ASO into cultured muscle cells obtained from a patient with DMD with a deletion of exon 20 ^(14)^. Subsequently, the patient received the ASO via intravenous infusion once a week for 4 weeks ^(15)^, and a muscle biopsy performed at 1 week after the final infusion revealed a novel in-frame mRNA lacking both exon 19 and exon 20 that represented approximately 6% of the total reverse transcription PCR product. Dystrophin was identified histochemically in the sarcolemma of muscle cells after ASO treatment. These findings demonstrate that ASO-mediated exon skipping shows promise for the treatment of DMD.

### 3-4. Approval of ASOs for DMD

The *DMD* gene contains numerous hotspots for exon deletion. Among exon deletions in patients with DMD, approximately 20%, 13%, 12%, and 11% are amendable with the skipping of exons 51, 53, 45, and 44, respectively ^(16)^. To treat a sufficient number of patients with DMD, several ASOs targeting the top three exons have been developed for clinical use by international researchers. As a result, eteplirsen, a 25-mer phosphorodiamidate morpholino ASO injected intravenously, was the first approved by the Food and Drug Administration (FDA) in 2016 for the treatment of patients with DMD with mutations amenable to the skipping of exon 51 (Table 1). Golodirsen, a morpholino ASO targeting the skipping of exon 53, was next approved in 2019 ^(17)^, and viltolarsen, a 21-mer phosphorodiamidate morpholino ASO targeting the skipping of exon 51, was approved in 2020 ^(18)^.

**Table 1.** Antisense Oligonucleotides (ASOs) to Treat Duchenne Muscular Dystrophy (DMD).

Name	ASO			Target exon	Phase
	Nucleic acid	Size	Route		
**Eteplirsen**	PMO	25	IV	51	Approved
**Golodirsen**	PMO	25	IV	53	Approved
**Viltolarsen**	PMO	21	IV	53	Approved
**Casimersen**	PMO	25	IV	45	Approval stage
**Renadirsen**	ENA/2'RNA	18	SC	45	I/II

PMO: phosphorodiamidate morpholino; ENA: 2'-O,4'-C-ethylene-bridged nucleic acid; 2'RNA: 2'-O-methyl RNA; IV: intravenous injection; SC: subcutaneous injection

We developed an 18-mer ASO that induces the skipping of exon 45, named renadirsen, that contains both 2'-O,4'-C-ethylene-bridged nucleic acid (ENA) and 2'-O-methyl RNA ^(19)^. A modified ENA invented in Japan was shown to have suitable characteristics for clinical use, such as high affinity for complementary sequences and high resistance to nuclease ^(20)^. Renadirsen was injected subcutaneously into patients in a phase I/II study (JapicCTI-153072).

## 4. Applications of Exon-skipping Therapies

ASO-mediated exon skipping serves as a method to edit mRNA and can therefore be applied to manipulate gene functions for restoration, destruction, and modulation (Table 2). For the restoration of gene function, mRNAs that lose certain exons should be functional or semifunctional, and this strategy is applied mainly to induce the skipping of mutated exons in monogenic diseases. In contrast, ASO-mediated exon skipping is applied for the disruption of gene function, which involves the targeting of out-of-frame exons, and this strategy has been applied to suppress *MSTN* gene function, thereby promoting muscle growth. In particular, the skipping of in-frame exons has been implemented to modulate gene function, producing an isoform that differs in structure and function from the original product. This strategy has also been applied to the removal of an integrin alpha 4 transmembrane domain by the skipping of exon 27 of the *ITG4* gene for the treatment of Crohn’s disease. Although exon skipping has multiple potential applications, caution should be taken to avoid the unintentional skipping of flanking exons, which has been reported in the *ITGA4* and* COL7A1* genes ^(21), (22)^.

**Table 2.** Gene Functional Changes Induced by Exon Skipping.

Function	Target exon	Gene	Inheritance	Disease
Restoration	Normal out-of-frame	*DMD*	Monogenetic	DMD
	Mutated in-frame exon	*DYSF*	Monogenetic	LGMD2B
	Mutated out-of-frame exon	*SGCG*	Monogenetic	LGMD2C
	Mutated pseudo exon	*CLRN1*	Monogenetic	USH3
Destruction	Out-of-frame exon	*MSTN*	Non monogenetic	Diabetes mellitus
Modulation	Normal in-frame exon	*ITGA4*	Non monogenetic	Crohn’s disease

DMD: Duchenne muscular dystrophy; LGMD2B: limb girdle muscular dystrophy 2B; LGMD2C: limb girdle muscular dystrophy 2C; USH3: Usher syndrome type III

### 4-1. Skipping of out-of-frame exons for the restoration of gene function

**Figure 1.**
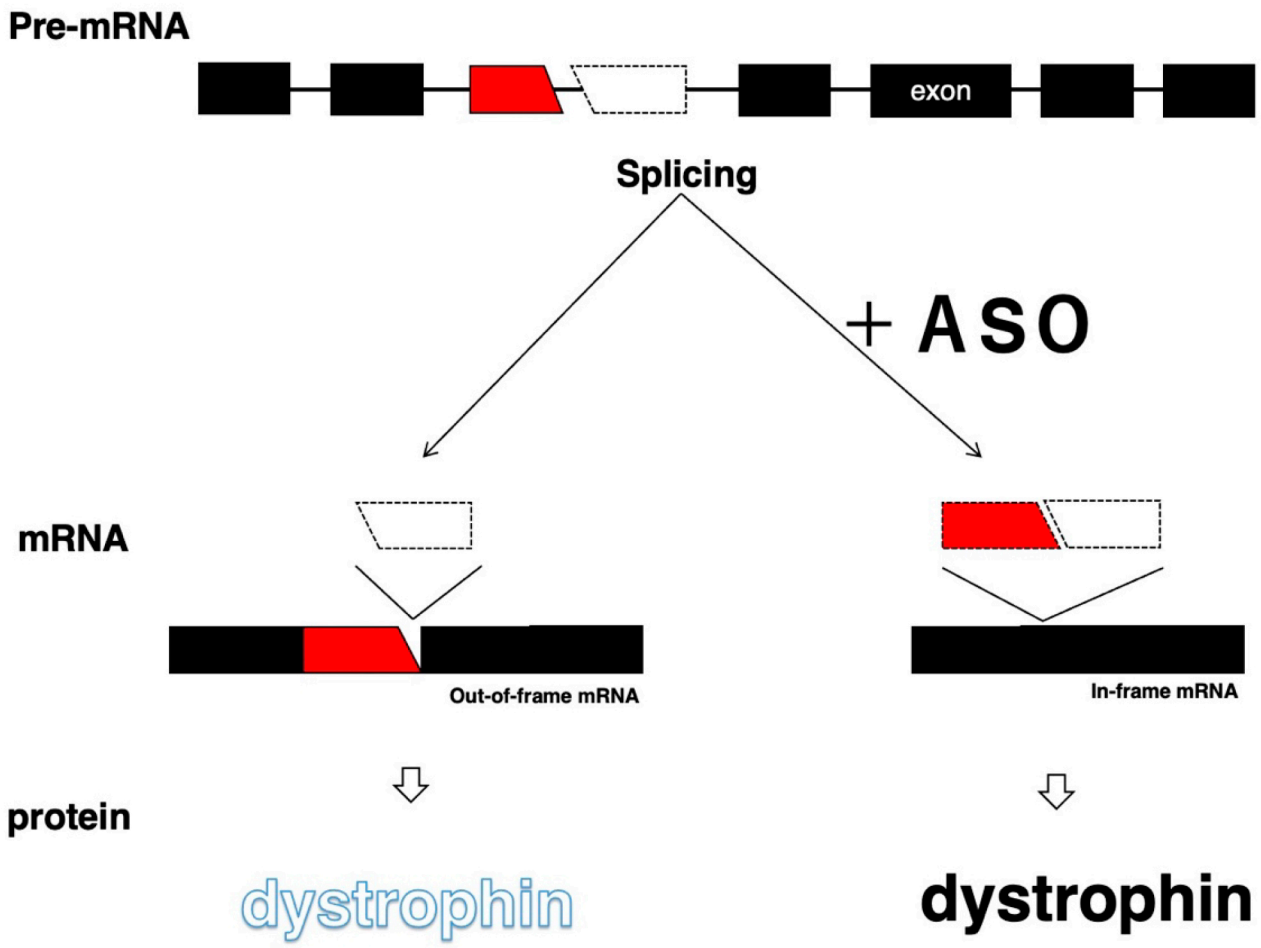
Antisense oligonucleotide (ASO)-mediated exon-skipping therapy. In DMD, an out-of-frame exon is deleted from the pre-mRNA (dotted box at the top). Therefore, the splicing produces an out-of-frame mRNA (middle) and no dystrophin (bottom). An ASO induces the skipping of the flanking exon to the deleted exon (red box) during splicing and produces an in-frame mRNA (middle), and the in-frame mRNA produces semifunctional dystrophin (bottom). The black boxes and lines indicate exons and introns, respectively.

The skipping of out-of-frame exons shifts the translational reading frame and is therefore applied to the conversion of out-of-frame mutations to in-frame mutations, as shown in DMD therapy (Figure 1 and Table 3). For dysferlinopathy and Parkinson’s disease, ASOs were designed for the skipping of *DYSF* and* PRKN* exons to restore the reading frame for the production of semifunctional proteins ^(23), (24)^.

**Table 3.** Skipping of Normal Out-of-frame Exons.

Disease	Gene	Exon	Phase	Author, Year
DMD	*DMD*	51, 53, 45	A, W, I/II	Schneider et al. 2021 ^(25)^
Dysferlinopathy	*DYSF*	51	P	Verwey et al. 2020 ^(23)^
Parkinson’s disease	*PRKN*	4	P	Li et al. 2020 ^(24)^

DMD: Duchenne muscular dystrophy; A: approved; W: wait for approval; P: preclinical study

### 4-2. Skipping of mutated in-frame exons for the restoration of gene function

The skipping of an in-frame exon encoding a disease-causing mutation aims to remove the mutation and produce in-frame mRNA, which should give rise to the semifunctional truncated protein. Therefore, this method can be applied to cases in which the truncated protein is confirmed to be semifunctional (Table 4). ASOs targeting mutated exons of the *DYSF* and *LMNA* genes have been studied to restore dysferlin in limb girdle muscular dystrophy 2B and laminin in laminopathy, respectively ^(26), (27)^. As most collagen molecules are synthesized in a trimeric procollagen form, exon-skipping therapies producing truncated proteins is challenging for the treatment of collagenopathy ^(28)^. Despite this, an ASO targeting exon 21 encoding a nonsense mutation of the *COL4A5* gene successfully improved the clinical phenotypes of a mouse model of Alport syndrome ^(29)^, suggesting that mutations in the in-frame exons of collagen genes are amendable by exon skipping. At the same time, an ASO designed to induce the skipping of exon 73 of the *COL7A1* gene, a mutational hotspot, was developed for the treatment of dystrophic epidermolysis bullosa ^(30)^. For dystrophic epidermolysis bullosa, ASOs can be administered topically, opening a new avenue for ASO transfection ^(30)^. ASO-mediated exon-skipping therapies have also been applied to several other diseases, including enzyme deficiency (Table 4).

**Table 4.** Skipping of Mutated In-frame Exons.

Disease	Gene	Exon	Phase	Author, Year
LGMD2B	*DYSF*	32	P	Barthelemy et al. 2015 ^(26)^
Laminopathy	*LMNA*	5	P	Scharner et al. 2015 ^(27)^
Joubert syndrome	*CEP290*	41	P	Molinari et al. 2019 ^(31)^
Alport syndrome	*COL4A5*	21	P	Yamamura et al. 2020 ^(29)^
Dystrophic epidermolysis bullosa	*COL7A1*	73	P	Bornert et al. 2020 ^(30)^
CADASIL	*NOTCH3*	9	P	Gravesteijn et al. 2020 ^(32)^
Mucolipidosis II	*GNPTAB*	19	P	Matos et al. 2020 ^(33)^
Myotonic dystrophy type1	*DMPK*	15	P	Stepniak et al. 2020 ^(34)^

LGMD2B: limb girdle muscular dystrophy 2B; CADASIL: cerebral autosomal dominant arteriopathy with subcortical infarcts and leukoencephalopathy; P: preclinical study

### 4-3. Skipping of mutated out-of-frame exons for the recovery of gene function

The skipping of mutated out-of-frame exons can remove the mutations but produce out-of-frame mRNAs incapable of producing proteins. Therefore, this strategy is applied in three situations: (1) the skipping of multiple exons to produce in-frame mRNA; (2) the skipping of a single exon to reduce a certain isoform; and (3) the skipping of the penultimate exon (Table 5). For example, four exons of the *SGCG* gene, including an out-of-frame exon with a mutation, were removed together using a combination of ASOs for the treatment of limb girdle muscular dystrophy type 2C ^(35)^. The skipping of an exon to reduce a certain isoform was applied in the treatment of laminopathy, which reduced laminin A production and thus led to the reduced accumulation of a toxic substance ^(36)^. The skipping of the penultimate exon has been investigated for the treatment of spinocerebellar ataxia type 3 by skipping exon 10 of the *ATXN3* gene ^(37)^.

**Table 5.** Skipping of Mutated Out-of-frame Exons.

Disease	Gene	Exon	Phase	Author, Year
LGMD2C	*SGCG*	4-7	P	Wyatt et al. 2018 ^(35)^
CADASIL	*NOTCH3*	2-3,4-5,6	P	Rutten 2016 ^(38)^
HGPS	*LMNA*	11	P	Lee et al. 2016 ^(36)^
SCA3	*ATXN3*	10	P	McIntosh et al. 2019 ^(37)^

LGMD2C: limb girdle muscular dystrophy 2C; CADASIL: cerebral autosomal dominant arteriopathy with subcortical infarcts and leukoencephalopathy; HPGS: Hutchinson-Gilford progeria syndrome; SCA3: spinocerebellar ataxia type 3; P: preclinical study

### 4-4. Skipping of mutated pseudoexons for the restoration of normal gene function

The skipping of mutated pseudoexons is expected to produce completely normal mRNAs and proteins and is therefore considered a complete treatment. One study revealed that pseudoexons were incorporated by the creation of 5' and 3' splice sites in 52 and 11 genes, respectively, and by the interference of splicing regulatory elements in nine genes ^(39)^. In cancer, pseudoexons have been identified in 10 genes directly involved in cancer pathology, such as *ATM*,* BRCA1*, and *NF1*, and in 32 cancer-related genes, such as *CYBB* and *CYP17A1* ^(40)^. Every pseudoexon can be a target of skipping therapy, and recent studies on pseudoexon skipping are summarized in Table 6. Nevertheless, mutations that create identical pseudoexons are rarely reported, and the low patient numbers have hampered the clinical development of ASOs designed for this purpose.

**Table 6.** Skipping of Pseudoexons.

Disease	Gene	Phase	Author, Year
Usher syndrome type III	*CLRN1*	P	Panagiotopoulos et al. 2020 ^(41)^
LGMD2B	*DYSF*	P	Dominov et al. 2020 ^(42)^
Erythropoietic protoporphyria	*FECH*	P	Halloy et al. 2020 ^(43)^
Stargardt disease	*ABCA4*	P	Sangermano et al. 2019 ^(44)^
Neuronal ceroid lipofuscinosis 7	*CLN7*	H	Kim et al. 2019 ^(45)^

LGMD2B: limb girdle muscular dystrophy 2B; P: preclinical study; H: human trial

### 4-5. Skipping of out-of-frame exons for the disruption of gene function

The skipping of out-of-frame exons is a gene silencing method that involves the alteration of the reading frame and has therefore been applied to treat diseases involving modifier genes (Table 7). As gene function can be silenced via techniques such as ASO-mediated RNase and siRNA transfection ^(46)^, ASO-mediated exon skipping has not been well studied for the treatment of diseases. An ASO designed to induce the skipping of the out-of-frame exon 2 of the *MSTN* gene was studied to enhance muscle growth in patients with muscular dystrophies and diabetes mellitus ^(47)^. An ASO designed to induce the skipping of exon 41 of *LRRK2*, which encodes part of the kinase domain, was developed to reduce LRRK2 kinase function in patients with genetic and sporadic Parkinson’s disease ^(48)^. Interestingly, an ASO designed to induce the skipping of the out-of-frame exon 4 of the fusion gene *ERG* was developed to suppress its function in prostate cancer cells and markedly reduced ERG protein levels, resulting in decreased cell proliferation ^(49)^.

**Table 7.** Skipping of Out-of-frame Exons for the Disruption of Gene Function.

Disease	Gene	Exon	Phase	Author, Year
Diabetes mellitus	*MSTN*	2	P	Eilers et al. 2021 ^(50)^
DMD			P	Kemaladewi et al. 2011 ^(47)^
Parkinson’s disease	*LRRK2*	41	P	Korecka et al. 2020 ^(48)^
Prostate cancer	*ERG*	4	P	Li et al. 2020 ^(49)^
Muscle fibrosis	*ALK5*	6	P	Kemaladewi et al. 2014 ^(51)^
Familial hypercholesterolemia	*APOB*	27	P	Disterer et al. 2013 ^(52)^

DMD: Duchenne muscular dystrophy; P: preclinical study

### 4-6. Skipping of normal in-frame exons for the modulation of gene function

**Figure 2.**
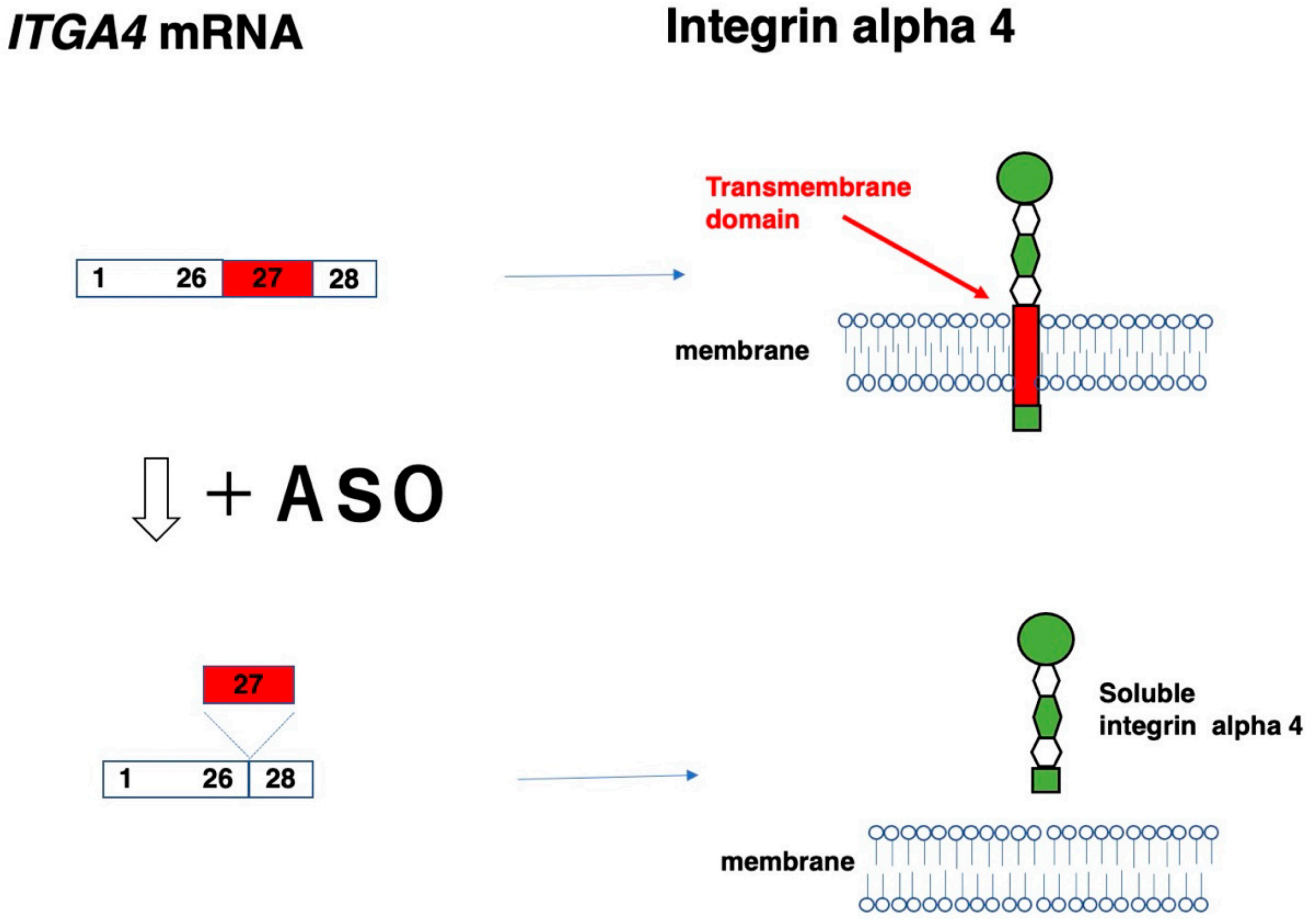
Production of soluble integrin alpha 4 by exon skipping. Integrin alpha 4 is encoded by the *ITGA4* gene, which produces an mRNA consisting of 28 exons (upper left). Integrin alpha 4 is localized to the membrane (upper right) through a transmembrane domain that is encoded in exon 27 (upper left). The ASO-induced skipping of exon 27 yields an exon 27-deleted mRNA (lower left) that produces an integrin alpha 4 protein with a deleted transmembrane domain in a soluble form (lower right). The box and number in the box indicate the exon and its number.

The skipping of in-frame exons produces in-frame mRNAs, thereby resulting in the production of short, truncated proteins. In cases of truncated proteins functioning differently from the original protein products, this exon skipping is applied to modulate gene function (Table 8). One example was reported in the *ITGA4* gene ^(21)^. Moreover, exon 27 was removed from the normal gene to produce an integrin alpha 4 protein lacking a transmembrane domain (Figure 2), resulting in the production of soluble integrin alpha 4 that bound beta. This strategy was devised to treat diseases such as Crohn’s disease and multiple sclerosis, which are modified by the hyperactivity of integrin beta.

**Table 8.** Skipping of Normal In-frame Exons.

Disease	Gene	Exon	Lost domain	Phase	Author, Year
Crohn’s disease	*ITGA4*	27	transmembrane	P	Aung-Htut 2019 ^(21)^
Dupuytren’s disease	*ALK5*	2	ligand binding	P	Karkampouna et al. 2014 ^(53)^
Muscle fibrosis				P	Kemaladewi et al. 2014 ^(51)^
Hypertrophic scar				P	Raktoe et al. 2020 ^(54)^
Cancer	*CD44*	v8	ligand binding	P	Fukushima et al. 2020 ^(55)^

P: preclinical study

## 5. Future Perspectives on ASO-mediated Exon-skipping Therapies: One Drug for One Patient

ASO-mediated exon-skipping therapies are now applicable for a wide variety of diseases. Currently, most exon-skippable ASOs under clinical development have been developed to treat a certain number of patients, and ASOs designed to induce the skipping of exons 51, 53, and 45 have been developed for the clinical treatment of patients with DMD. In contrast, patients with deletions not treatable with these ASOs do not benefit from these treatment opportunities, even though exon skipping is theoretically applicable. Recently, one ASO was developed for the treatment of one patient within a remarkably short time period to stop the aggressive progression of a neurodegenerative disease (Table 6) ^(45)^. This event stimulated a large discussion on precision medicine among staff at the FDA ^(56), (57)^, and the one-drug/one-patient policy may enhance the further development of ASOs and open a new avenue for the treatment of patients, especially those with rare uncurable diseases.

ASO-mediated exon-skipping therapy is an emerging area of drug development that targets the disease source at the RNA level and offers a promising therapy for a wide variety of diseases. However, there are some concerns about the therapy: first, side effects including off-target effects should be monitored carefully, because ultra-long-term treatment has not yet been completed. Second, currently approved ASOs are expensive. These high costs are not specific to ASOs but relate to the fact that ASOs mainly target rare diseases. As the number of treated patients is lower, development costs per patient are higher ^(58)^. The cost to synthesize ASO would be reduced by a large-scale synthesis when the demand for ASOs increases by treating common diseases. Third, the delivery of ASOs has always been a significant hurdle for their broad clinical applications, and various strategies have been employed to deliver them ^(59)^. For example, the conjugation of ASO to N-acetyl galactosamine (GalNAc) is a well-defined liver-targeted moiety, because GalNAc selectively targets the asialoglycoprotein receptor that is highly expressed in hepatocytes ^(60)^. These concerns will be solved by conducting more studies on ASO drugs in the future.

## Article Information

### Conflicts of Interest

Masafumi Matsuo discloses being employed by Kobe Gakuin University, which received funding from KNC Laboratories Inc. (Kobe, Japan). The author further discloses being a scientific adviser for Daiichi-Sankyo Co. (Tokyo, Japan) and JCR Pharma Co. (Ashiya, Japan).

### Sources of Funding

This work was partly supported by the Practical Research Project for Rare/Intractable Diseases from the Japan Agency for Medical Research and Development, AMED grant number [20ek0109442h 0001].

## References

[1] Takeshima Y, Nishio H, Sakamoto H, et al. Modulation of in vitro splicing of the upstream intron by modifying an intra-exon sequence which is deleted from the dystrophin gene in dystrophin Kobe. J Clin Invest. 1995;95(2):515-20. 7860733 10.1172/JCI117693 PMC295503

[2] Matsuo M. Duchenne/Becker muscular dystrophy: from molecular diagnosis to gene therapy. Brain Dev. 1996;18(3):167-72. 8836495 10.1016/0387-7604(96)00007-1

[3] Pan Q, Shai O, Lee LJ, et al. Deep surveying of alternative splicing complexity in the human transcriptome by high-throughput sequencing. Nat Genet. 2008;40(12):1413-5. 18978789 10.1038/ng.259

[4] Kim P, Yang M, Yiya K, et al. ExonSkipDB: functional annotation of exon skipping event in human. Nucleic Acids Res. 2020;48(D1):D896-907. 31642488 10.1093/nar/gkz917 PMC7145592

[5] Scotti MM, Swanson MS. RNA mis-splicing in disease. Nat Rev Genet. 2016;17(1):19-32. 26593421 10.1038/nrg.2015.3 PMC5993438

[6] Eagle M, Baudouin SV, Chandler C, et al. Survival in Duchenne muscular dystrophy: improvements in life expectancy since 1967 and the impact of home nocturnal ventilation. Neuromuscul Disord. 2002;12(10):926-9. 12467747 10.1016/s0960-8966(02)00140-2

[7] Passamano L, Taglia A, Palladino A, et al. Improvement of survival in Duchenne muscular dystrophy: retrospective analysis of 835 patients. Acta Myol. 2012;31(2):121-5. 23097603 PMC3476854

[8] Koenig M, Hoffman EP, Bertelson CJ, et al. Complete cloning of the Duchenne muscular dystrophy (DMD) cDNA and preliminary genomic organization of the DMD gene in normal and affected individuals. Cell. 1987;50(3):509-17. 3607877 10.1016/0092-8674(87)90504-6

[9] Monaco AP, Bertelson CJ, Liechti-Gallati S, et al. An explanation for the phenotypic differences between patients bearing partial deletions of the DMD locus. Genomics. 1988;2(1):90-5. 3384440 10.1016/0888-7543(88)90113-9

[10] Matsuo M, Takeshima Y, Nishio H. Contributions of Japanese patients to development of antisense therapy for DMD. Brain Dev. 2016;38(1):4-9. 26094594 10.1016/j.braindev.2015.05.014

[11] Matsuo M, Masumura T, Nakajima T, et al. A very small frame-shifting deletion within exon 19 of the Duchenne muscular dystrophy gene. Biochem Biophys Res Commun. 1990;170(2):963-7. 2383276 10.1016/0006-291x(90)92185-3

[12] Pramono ZA, Takeshima Y, Alimsardjono H, et al. Induction of exon skipping of the dystrophin transcript in lymphoblastoid cells by transfecting an antisense oligodeoxynucleotide complementary to an exon recognition sequence. Biochem Biophys Res Commun. 1996;226(2):445-9. 8806654 10.1006/bbrc.1996.1375

[13] Shiga N, Takeshima Y, Sakamoto H, et al. Disruption of the splicing enhancer sequence within exon 27 of the dystrophin gene by a nonsense mutation induces partial skipping of the exon and is responsible for Becker muscular dystrophy. J Clin Invest. 1997;100(9):2204-10. 9410897 10.1172/JCI119757 PMC508415

[14] Takeshima Y, Wada H, Yagi M, et al. Oligonucleotides against a splicing enhancer sequence led to dystrophin production in muscle cells from a Duchenne muscular dystrophy patient. Brain Dev. 2001;23(8):788-90. 11720794 10.1016/s0387-7604(01)00326-6

[15] Takeshima Y, Yagi M, Wada H, et al. Intravenous infusion of an antisense oligonucleotide results in exon skipping in muscle dystrophin mRNA of Duchenne muscular dystrophy. Pediatr Res. 2006;59(5):690-4. 16627883 10.1203/01.pdr.0000215047.51278.7c

[16] Flotats BM, Hahn A. New therapeutics options for pediatric neuromuscular disorders. Front Pediatr. 2020;8:583877. 33330280 10.3389/fped.2020.583877 PMC7719776

[17] Heo YA. Golodirsen: first approval. Drugs. 2020;80(3):329-33. 32026421 10.1007/s40265-020-01267-2

[18] Komaki H, Takeshima Y, Matsumura T, et al. Viltolarsen in Japanese Duchenne muscular dystrophy patients: a phase 1/2 study. Ann ClinTransl Neurol. 2020;7(12):2393-408. 10.1002/acn3.51235 PMC7732240 33285037

[19] Lee T, Awano H, Yagi M, et al. 2'-O-methyl RNA/ethylene-bridged nucleic acid chimera antisense oligonucleotides to induce dystrophin exon 45 skipping. Genes. 2017;8(2):67. 10.3390/genes8020067 PMC5333056 28208626

[20] Koizumi M. 2'-O,4'-C-ethylene-bridged nucleic acids (ENA) as next-generation antisense and antigene agents. Biol Pharm Bull. 2004;27(4):453-6. 15056846 10.1248/bpb.27.453

[21] Aung HM, Comerford I, Johnsen R, et al. Reduction of integrin alpha 4 activity through splice modulating antisense oligonucleotides. Sci Rep. 2019;9:12994. 31506448 10.1038/s41598-019-49385-6 PMC6736852

[22] Ham KA, Aung-Htut MT, Fletcher S, et al. Nonsequential splicing events alter antisense-mediated exon skipping outcome in COL7A1. Int J Mol Sci. 2020;21(20):7705. 10.3390/ijms21207705 PMC7590164 33081018

[23] Verwey N, Gazzoli I, Krause S, et al. Antisense-mediated skipping of dysferlin exons in control and dysferlinopathy patient-derived cells. Nucleic Acid Ther. 2020;30(21):71-9. 31873062 10.1089/nat.2019.0788

[24] Li D, Aung-Htut MT, Ham KA, et al. A Splice intervention therapy for autosomal recessive juvenile Parkinson's disease arising from parkin mutations. Int J Mol Sci. 2020;21(19):7282. 10.3390/ijms21197282 PMC7582384 33019779

[25] Schneider AF, Aartsma RA. Developments in reading frame restoring therapy approaches for Duchenne muscular dystrophy. Expert Opin Biol Ther. 2021;21(3):343-59. 33074029 10.1080/14712598.2021.1832462

[26] Barthélémy F, Blouin C, Wein N, et al. Exon 32 skipping of dysferlin rescues membrane repair in patients' cells. J Neuromuscul Dis. 2015;2(3):281-90. 27858744 10.3233/JND-150109 PMC5240545

[27] Scharner J, Figeac N, Ellis JA, et al. Ameliorating pathogenesis by removing an exon containing a missense mutation: a potential exon-skipping therapy for laminopathies. Gene Ther. 2015;22(6):503-15. 25832542 10.1038/gt.2015.8

[28] Li D, Mastaglia F, Fletcher S, et al. Precision medicine through antisense oligonucleotide-mediated exon skipping. Trends Pharmacol Sci. 2018;39(11):982-94. 30282590 10.1016/j.tips.2018.09.001

[29] Yamamura T, Horinouchi T, Adachi T, et al. Development of an exon skipping therapy for X-linked Alport syndrome with truncating variants in COL4A5. Nat Commun. 2020;11(1):2777. 32488001 10.1038/s41467-020-16605-x PMC7265383

[30] Bornert O, Hogervorst M, Nauroy P, et al. QR-313, an antisense oligonucleotide, shows therapeutic efficacy for treatment of dominant and recessive dystrophic epidermolysis bullosa: a preclinical study. J Invest Dermatol. 2021;141(4):883-93.e6. 32946877 10.1016/j.jid.2020.08.018

[31] Molinari E, Ramsbottom S, Srivastava S, et al. Targeted exon skipping rescues ciliary protein composition defects in Joubert syndrome patient fibroblasts. Sci Rep. 2019;9(1):10828. 31346239 10.1038/s41598-019-47243-z PMC6658666

[32] Gravesteijn G, Dauwerse J, Overzier M, et al. Naturally occurring NOTCH3 exon skipping attenuates NOTCH3 protein aggregation and disease severity in CADASIL patients. Hum Mol Genet. 2020;29(11):1853-63. 31960911 10.1093/hmg/ddz285 PMC7372551

[33] Matos L, Vilela R, Rocha M, et al. Development of an antisense oligonucleotide-mediated exon skipping therapeutic strategy for mucolipidosis II: validation at RNA level. Hum Gene Ther. 2020;31(13-14):775-83. 32283951 10.1089/hum.2020.034

[34] Stepniak-Konieczna E, Konieczny P, Cywoniuk P, et al. AON-induced splice-switching and DMPK pre-mRNA degradation as potential therapeutic approaches for Myotonic Dystrophy type 1. Nucleic Acids Res. 2020;48(5):2531-43. 31965181 10.1093/nar/gkaa007 PMC7049696

[35] Wyatt EJ, Demonbreun AR, Kim EY, et al. Efficient exon skipping of SGCG mutations mediated by phosphorodiamidate morpholino oligomers. JCI Insight. 2018;3(9):e99357. 10.1172/jci.insight.99357 PMC6012523 29720576

[36] Lee JM, Nobumori C, Tu Y, et al. Modulation of LMNA splicing as a strategy to treat prelamin A diseases. J Clin Invest. 2016;126(4):1592-602. 26999604 10.1172/JCI85908 PMC4811112

[37] McIntosh CS, Aung-Htut MT, Fletcher S, et al. Removal of the polyglutamine repeat of ataxin-3 by redirecting pre-mRNA processing. Int J Mol Sci. 2019;20(21):5434. 10.3390/ijms20215434 PMC6862616 31683630

[38] Rutten JW, Dauwerse HG, Peters DJM, et al. Therapeutic NOTCH3 cysteine correction in CADASIL using exon skipping: in vitro proof of concept. Brain. 2016;139(4):1123-35. 26912635 10.1093/brain/aww011

[39] Vaz-Drago R, Custódio N, Carmo-Fonseca M. Deep intronic mutations and human disease. Hum Genet. 2017;136(9):1093-111. 28497172 10.1007/s00439-017-1809-4

[40] Romano M, Buratti E, Baralle D. Role of pseudoexons and pseudointrons in human cancer. Int J Cell Biol. 2013;2013:810572. 24204383 10.1155/2013/810572 PMC3800588

[41] Panagiotopoulos AL, Karguth N, Pavlou M, et al. Antisense oligonucleotide- and CRISPR-Cas9-mediated rescue of mRNA splicing for a deep intronic CLRN1 mutation. Mol Ther Nucleic Acids. 2020;21:1050-61. 32841912 10.1016/j.omtn.2020.07.036 PMC7452116

[42] Dominov J, Uyan Ö, McKenna-Yasek D, et al. Correction of pseudoexon splicing caused by a novel intronic dysferlin mutation. Ann Clin Transl Neurol. 2019;6:642-54. 31019989 10.1002/acn3.738 PMC6469257

[43] Halloy F, Iyer P, Ćwiek P, et al. Delivery of oligonucleotides to bone marrow to modulate ferrochelatase splicing in a mouse model of erythropoietic protoporphyria. Nucleic Acids Res. 2020;48(9):4658-71. 32313951 10.1093/nar/gkaa229 PMC7229840

[44] Sangermano R, Garanto A, Runhart E, et al. Deep-intronic ABCA4 variants explain missing heritability in Stargardt disease and allow correction of splice defects by antisense oligonucleotides. Genet Med. 2019;21(8):1751-60. 30643219 10.1038/s41436-018-0414-9 PMC6752325

[45] Kim J, Hu C, Moufawad El Achkar C, et al. Patient-customized oligonucleotide therapy for a rare genetic disease. N Engl J Med. 2019;381(17):1644-52. 31597037 10.1056/NEJMoa1813279 PMC6961983

[46] Fuchs U, Damm-Welk C, Borkhardt A. Silencing of disease-related genes by small interfering RNAs. Curr Mol Med. 2004;4(5):507-17. 15267222 10.2174/1566524043360492

[47] Kemaladewi DU, Hoogaars WM, van Heiningen SH, et al. Dual exon skipping in myostatin and dystrophin for Duchenne muscular dystrophy. BMC Med Genomics. 2011;4(1):36. 21507246 10.1186/1755-8794-4-36 PMC3107769

[48] Korecka JA, Thomas R, Hinrich AJ, et al. Splice-switching antisense oligonucleotides reduce LRRK2 kinase activity in human LRRK2 transgenic mice. Mol Ther Nucleic Acids. 2020;21:623-35. 32736291 10.1016/j.omtn.2020.06.027 PMC7393423

[49] Li L, Hobson L, Perry L, et al. Targeting the ERG oncogene with splice-switching oligonucleotides as a novel therapeutic strategy in prostate cancer. Brit J Cancer. 2020;123(6):1024-32. 32581342 10.1038/s41416-020-0951-2 PMC7493922

[50] Eilers W, Cleasby M, Foster K. Development of antisense-mediated myostatin knockdown for the treatment of insulin resistance. Sci Rep. 2021;11(1):1604. 33452345 10.1038/s41598-021-81222-7 PMC7810755

[51] Kemaladewi D, Pasteuning S, van der Meulen JW, et al. Targeting TGF-β signaling by antisense oligonucleotide-mediated knockdown of TGF-β type I receptor. Mol Ther Nucleic Acids. 2014;3:e156. 24691207 10.1038/mtna.2014.7 PMC4011125

[52] Disterer P, Al Shawi R, Ellmerich S, et al. Exon skipping of hepatic APOB pre-mRNA with splice-switching oligonucleotides reduces LDL cholesterol in vivo. Mol Ther. 2013;21(3):602-9. 23319054 10.1038/mt.2012.264 PMC3589156

[53] Karkampouna S, Kruithof B, Kloen P, et al. Novel ex vivo culture method for the study of Dupuytren's disease: effects of TGFβ type 1 receptor modulation by antisense oligonucleotides. Mol Ther Nucleic Acids. 2014;3:e142. 24448195 10.1038/mtna.2013.69 PMC3912325

[54] Raktoe RS, Rietveld MH, Out-Luiting J, et al. Exon skipping of TGFβRI affects signalling and ECM expression in hypertrophic scar-derived fibroblasts. Scars Burn Heal. 2020;6:2059513120908857. 32528734 10.1177/2059513120908857 PMC7263111

[55] Fukushima S, Farea M, Maeta K, et al. Dual fluorescence splicing reporter minigene identifies an antisense oligonucleotide to skip exon v8 of the CD44 gene. Int J Mol Sci. 2020;21(23):E9316. 33266296 10.3390/ijms21239136 PMC7729581

[56] Fyfe I. Oligonucleotide designed for ultimate personalized treatment. Nat Rev Neurol. 2019;15(12):687. 31673092 10.1038/s41582-019-0286-x

[57] Woodcock J, Marks P. Drug regulation in the era of individualized therapies. N Engl J Med. 2019;381(17):1678-80. 31597016 10.1056/NEJMe1911295

[58] Kuijper E, Bergsma A, Pijnappel WWMP, et al. Opportunities and challenges for antisense oligonucleotide therapies. J Inherit Metab Dis. 2021;44(1):72-87. 32391605 10.1002/jimd.12251 PMC7891411

[59] Dhuri K, Bechtold C, Quijano E, et al. Antisense oligonucleotides: an emerging area in drug discovery and development. J Clin Med. 2020;9(6):2004. 10.3390/jcm9062004 PMC7355792 32604776

[60] Huang Y. Preclinical and clinical advances of GalNAc-decorated nucleic acid therapeutics. Mol Ther Nucleic Acids. 2017;6:116-32. 28325278 10.1016/j.omtn.2016.12.003 PMC5363494

